# Supplementary material for: GOPC:ROS1 and other ROS1 fusions represent a rare but recurrent drug target in a variety of glioma types
Source: Acta Neuropathol. 2021 Sep 18;142(6):1065–9. doi: 10.1007/s00401-021-02369-1 (PMC8568855; doi:10.1007/s00401-021-02369-1)
Supplement: Supplementary file 1 — Supplementary file1 (PDF 700 KB) [file 401_2021_2369_MOESM1_ESM.pdf]

***GOPC:ROS1* and other *ROS1* fusions represent a rare but recurrent drug target in a variety of glioma types**

Supplementary materials and methods

Sample collection

Tumor samples and patient data were obtained from multiple national and international collaborating centers and collected at the Department of Neuropathology of the University Hospital Heidelberg (Germany). Analysis of tissue and clinical data was performed in accordance with local ethics regulations. Clinical details of the patients are listed in Supplementary Table 1.

DNA methylation array processing and copy number profiling

Genomic DNA was extracted from fresh-frozen or formalin-fixed and paraffin-embedded (FFPE) tissue samples. Genome-wide DNA methylation profiling of all samples was performed using the Infinium MethylationEPIC (EPIC) BeadChip (Illumina, San Diego, CA, USA) or Infinium HumanMethylation450 (450k) BeadChip array (Illumina) according to the manufacturer's instructions and as previously described [2]. Raw data were generated at the Department of Neuropathology of the University Hospital Heidelberg, the Genomics and Proteomics Core Facility of the German Cancer Research Center (DKFZ) or at respective international collaborator institutes, using both fresh-frozen and formalin-fixed paraffin-embedded (FFPE) tissue samples. All computational analyses were performed in R version 3.6.0 (R Development Core Team, 2016; https://www.R-project.org). Copy-number variation analysis from 450k and EPIC methylation array data was performed using the conumee Bioconductor package version 1.12.0 [1]. Raw signal intensities were obtained from IDAT-files using the minfi Bioconductor package version 1.21.4. Illumina EPIC and 450k samples were merged to a combined data set by selecting the intersection of probes present on both arrays (combineArrays function, minfi). Each sample was individually normalized by performing a background correction (shifting of the 5% percentile of negative control probe intensities to 0) and a dye-bias correction (scaling of the mean of normalization control probe intensities to 10,000) for both color channels. Subsequently, a correction for the array type (450k/EPIC) was performed by fitting univariable, linear models to the log2-transformed intensity values (removeBatchEffect function, limma package version 3.30.11). The methylated and unmethylated signals were corrected individually. Beta-values were calculated from the retransformed intensities using an offset of 100 (as recommended by Illumina). All samples were checked for duplicates by pairwise correlation of the genotyping probes on the 450k/EPIC array. To perform unsupervised non-linear dimension reduction, the remaining probes after standard filtering [2] were used to calculate the 1-variance weighted Pearson correlation between samples. The resulting distance matrix was used as input for t-SNE analysis (t-distributed stochastic neighbor embedding; Rtsne package version 0.13). The following non-default parameters were applied: theta = 0, pca = F, max_iter = 10,000 perplexity = 20.

Automated screening and identification of *ROS1*-fused glioma samples using DNA methylation data

For identification of putative relevant cases showing a *ROS1* fusion we pre-analyzed DNA methylation data of 20,723 glioma samples as follows. As *ROS1* fusions are typically accompanied by a segmental loss of chromosome 6q22 in the copy-number profile, DNA methylation data from 20,723 glioma samples were screened for a segmental loss of this region. Cases with low quality copy-number profiles were filtered. Automated analysis was followed by visual inspection and led to identification of 14 cases with sufficient material for confirmation via RNA sequencing.

RNA sequencing and analysis

RNA was extracted from FFPE tissue samples using the automated Maxwell system with the Maxwell 16 LEV RNA FFPE Kit (Promega, Madison, WI, USA), according to the manufacturer's instructions. Transcriptome analysis using messenger RNA (mRNA) sequencing of samples was performed on a NextSeq 500 instrument (Illumina) as previously described [6]. Fastq files from transcriptome sequencing were used for *de novo* annotation of fusion transcripts using the defuse [4] and Arriba (v1.2.0) [7] algorithms with standard parameters. The fusion plot was created using the R-script for visualization provided in Arriba. All further analysis was performed in R (version 3.6.0; R Core Team, 2019) using the DESeq2 package (v1.28.1) [3].

DNA sequencing and mutational analysis

Capture-based next-generation DNA sequencing was performed on a NextSeq 500 instrument (Illumina) as previously described [5] using a custom brain tumor panel (Agilent Technologies, Santa Clara, CA, USA) covering the entire coding and selected intronic and promoter regions of 130 genes of particular relevance in central nervous system tumors.

References to supplementary methods

1. Aryee MJ, Jaffe AE, Corrada-Bravo H, Ladd-Acosta C, Feinberg AP, Hansen KD et al. (2014) Minfi: a flexible and comprehensive Bioconductor package for the analysis of Infinium DNA methylation microarrays. Bioinformatics 30:1363-1369. doi:10.1093/bioinformatics/btu049
2. Capper D, Jones DTW, Sill M, Hovestadt V, Schrimpf D, Sturm D et al. (2018) DNA methylation-based classification of central nervous system tumours. Nature 555:469-474. doi:10.1038/nature26000
3. Love MI, Huber W, Anders S (2014) Moderated estimation of fold change and dispersion for RNA-seq data with DESeq2. Genome Biol 15:550. doi:10.1186/s13059-014-0550-8
4. McPherson A, Hormozdiari F, Zayed A, Giuliany R, Ha G, Sun MG et al. (2011) deFuse: an algorithm for gene fusion discovery in tumor RNA-Seq data. PLoS Comput Biol 7:e1001138. doi:10.1371/journal.pcbi.1001138
5. Sahm F, Schrimpf D, Jones DT, Meyer J, Kratz A, Reuss D et al. (2016) Next-generation sequencing in routine brain tumor diagnostics enables an integrated diagnosis and identifies actionable targets. Acta Neuropathol 131:903-910. doi:10.1007/s00401-015-1519-8

6. Stichel D, Schrimpf D, Casalini B, Meyer J, Wefers AK, Sievers P et al. (2019) Routine RNA sequencing of formalin-fixed paraffin-embedded specimens in neuropathology diagnostics identifies diagnostically and therapeutically relevant gene fusions. Acta Neuropathol 138:827-835. doi:10.1007/s00401-019-02039-3
7. Uhrig S, Ellermann J, Walther T, Burkhardt P, Frohlich M, Hutter B et al. (2021) Accurate and efficient detection of gene fusions from RNA sequencing data. Genome Res 31:448-460. doi:10.1101/gr.257246.119

Supplementary Table 1 - Summary of clinicopathological characteristics and key genetic alterations

Case	Sex	Age (years)	Location	Histology	MC prediction v12	Gene fusion	Indication for a *ROS1* fusion by CNV	Additional CNV	NGS findings
#01	f	1	cerebellum, brain stem	LGG	LGG, PA PF	*GOPC:ROS1*	focal loss chr 6q	-	-
#02	m	6	parietal left	LGG/LGGNT	LGG, PA/GG	*GOPC:ROS1*	focal loss chr 6q	unclear baseline	-
#03	f	45	n/a	GBM	GBM, RTK II	*GOPC:ROS1*	focal loss chr 6q	gain chr 7, loss chr 10, gain chr 20, loss chr 22q, CDKN2A/B homozygous deletion, CDK4 amplification, MDM4 amplification	TERT promoter C250T<br>PTEN:NM_000314:exon2:c.G141T:p.R47S
#04	m	0	hemispheric left	GBM	IHG	*GOPC:ROS1*	focal loss chr 6q	CDKN2A/B homozygous deletion	-
#05	m	2	n/a	pO	nc	*GOPC:ROS1*	focal loss chr 6q	seg. loss chr 22q	-
#06	f	68	fronto-parietal left	GBM	GBM, RTK II	*GOPC:ROS1*	focal loss chr 6q	gain chr 7, loss chr 10, CDKN2A/B homozygous deletion	TERT promoter C250T<br>TP53:NM_001126116:exon1:c.G71C:p.R24P
#07	m	2	left ventricle	PMA	nc	*GOPC:ROS1*	focal loss chr 6q	seg. loss chr 19p	-
#08	f	12	thalamic right	GNT (high-grade)	nc	*GOPC:ROS1*	focal loss chr 6q	loss chr 1p, CDKN2A/B homozygous deletion	-
#09	f	0	hemispheric left	GBM	IHG	*GOPC:ROS1*	focal loss chr 6q	-	-
#10	m	7	right ventricle	PA	nc	*GOPC:ROS1*	focal loss chr 6q	gain chr 14q, loss chr 22q	-
#11	m	0	hemispheric right	AA	IHG	*GOPC:ROS1*	focal loss chr 6q	-	n/a
#12	f	2	medulla oblongata	HGG	nc	*GOPC:ROS1*	focal loss chr 6q	-	-
#13	m	2	fronto-parietal right	GBM	IHG	*ARCN1:ROS1*	focal loss chr 6q	loss chr 10, seg. loss chr 2q, 5p, 9p, 11q	PTEN:NM_000314:exon5:c.C388G:p.R130G
#14	m	0	fronto-parietal right	GBM	IHG	*CHCHD3:ROS1*	focal loss chr 6q	gain chr 11q, loss chr 6p	-
#15	m	2	temporo-parietal right	GBM	IHG	*ZCCHC8:ROS1*	focal loss chr 6q	gain chr 8	-
#16	m	16	posterior fossa	PA	nc	*CEP85L:ROS1*	focal gain chr 6q	-	-

Abbreviations: f, female; m, male; n/a, not available; LGG, low-grade glioma; LGGNT, low-grade glioneuronal tumor; GBM, glioblastoma; pO, pediatric oligodendroglioma; PMA, pilomyxoid astrocytoma; GNT, glioneuronal tumor (high-grade); AA, anaplastic astrocytoma; PA, pilocytic astrocytoma; MC, DNA methylation class; LGG, PA PF, posterior fossa pilocytic astrocytoma; LGG, PA/GG, hemispheric pilocytic astrocytoma and ganglioglioma; GBM, RTK II, glioblastoma IDH wildtype, subclass RTK II; IHG, infantile hemispheric glioma; nc, not classifiable; CNV, copy number variations; NGS, next-generation sequencing.

Supplementary Table 2 - Summary of the fusion detection algorithm output ‘Arriba’

case	gene1	gene2	breakpoint1	breakpoint2	site1	site2	split_reads1	split_reads2	discordant_mates	coverage1	coverage2	confidence	reading_frame
#01	*GOPC*	*ROS1*	6:117888017	6:117642557	splice-site	splice-site	8	0	7	49	19	medium	in-frame
#02	*GOPC*	*ROS1*	6:117888017	6:117642557	splice-site	splice-site	7	1	4	87	24	medium	in-frame
#03	*GOPC*	*ROS1*	6:117888017	6:117642557	splice-site	splice-site	22	2	9	167	54	high	in-frame
#04	*GOPC*	*ROS1*	6:117896313	6:117641184	intron	CDS	1	7	6	53	39	low	in-frame
#05	*GOPC*	*ROS1*	6:117888017	6:117642557	splice-site	splice-site	18	3	17	167	49	high	in-frame
#06	*GOPC*	*ROS1*	6:117888017	6:117642557	splice-site	splice-site	6	4	7	72	18	high	in-frame
#07	*GOPC*	*ROS1*	6:117896340	6:117641193	splice-site	splice-site	0	3	5	25	17	low	in-frame
#08	*GOPC*	*ROS1*	6:117888017	6:117642557	splice-site	splice-site	1	0	2	27	8	low	in-frame
#09	*GOPC*	*ROS1*	6:117896366	6:117641174	CDS	CDS	1	12	21	120	70	medium	in-frame
#10	*GOPC*	*ROS1*	6:117888017	6:117642557	splice-site	splice-site	8	4	6	112	27	high	in-frame
#11	*GOPC*	*ROS1*	6:117896340	6:117641193	splice-site	splice-site	1	4	4	33	17	low	in-frame
#13	*ARCN1*	*ROS1*	11:118455359	6:117641193	splice-site	splice-site	2	6	6	80	30	high	in-frame
#14	*CHCHD3*	*ROS1*	7:132719821	6:117641190	intron	CDS	3	0	2	93	168	medium	in-frame
#15	*ZCCHC8*	*ROS1*	12:122983374	6:117641193	splice-site	splice-site	2	14	6	80	46	high	in-frame

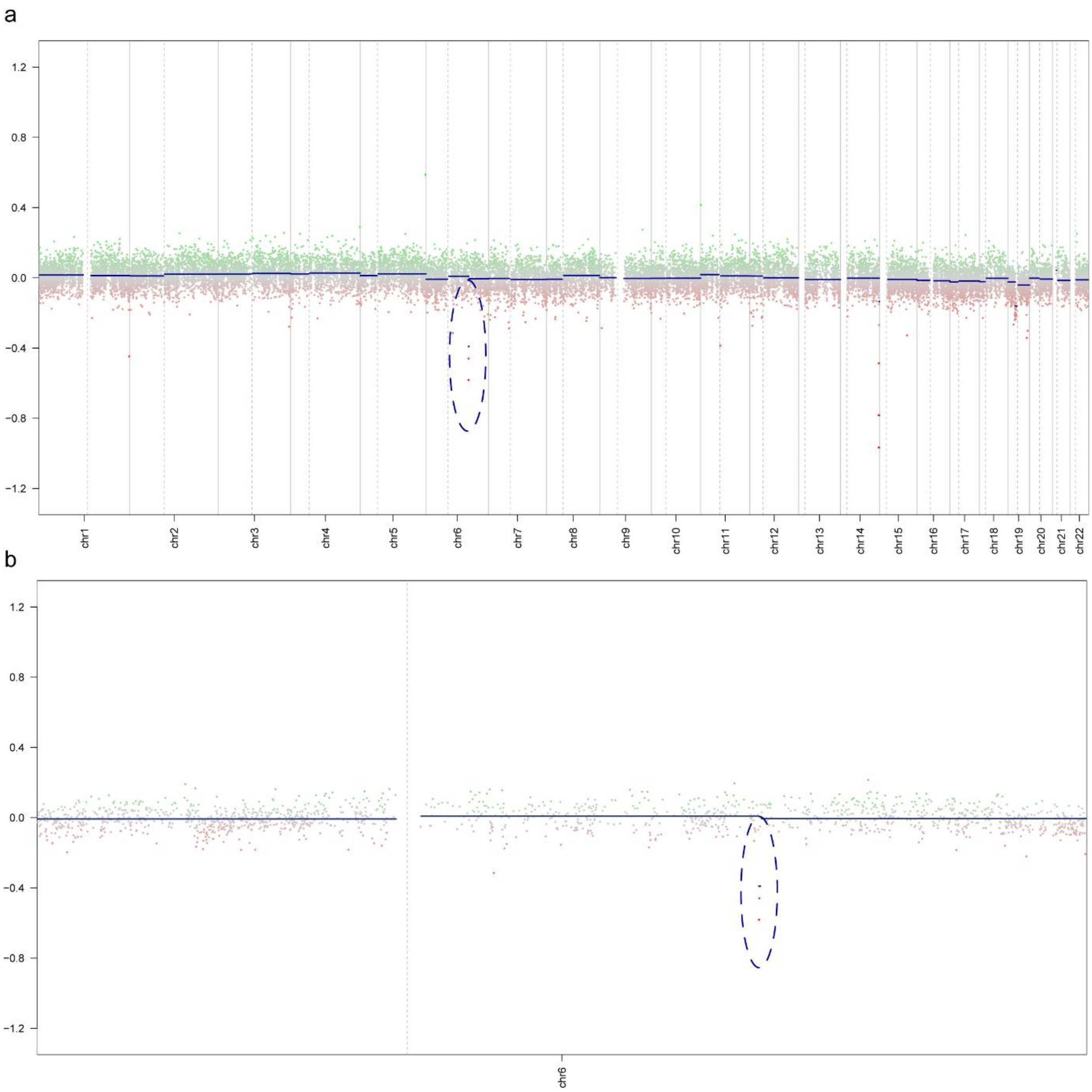

Supplementary Fig. 1 Copy-number profile derived from DNA methylation array data showing a focal loss of chromosome 6q22 around the *ROS1* locus (a). Enlarged view of chromosome 6 from the same case (b).

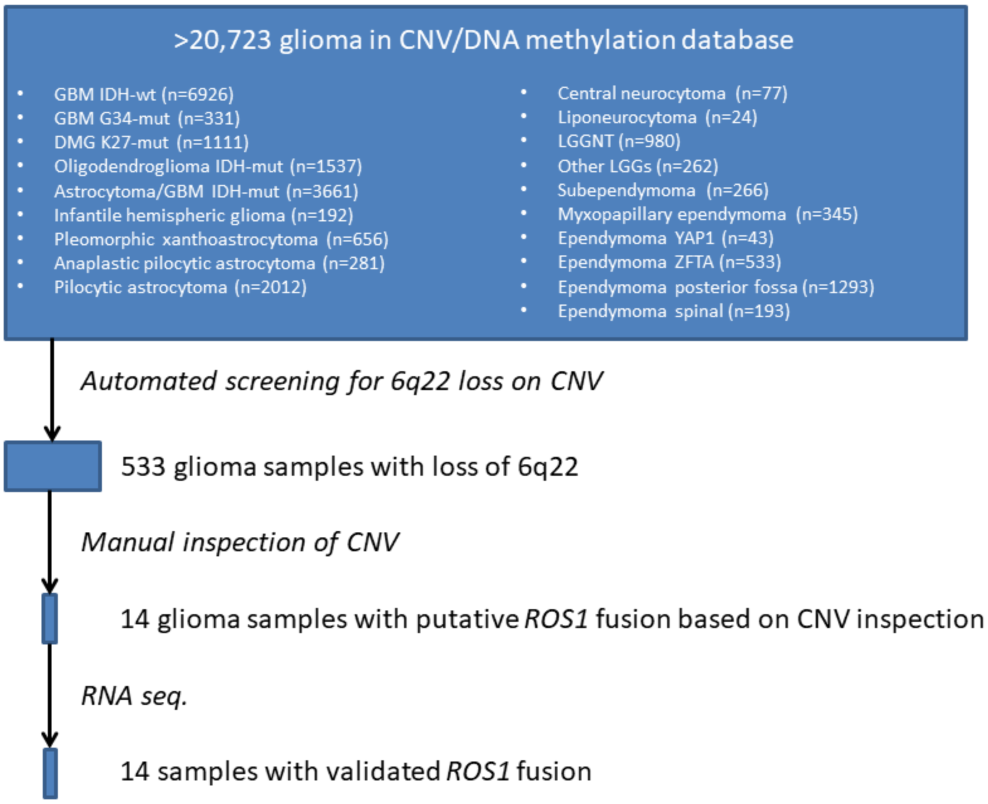

Supplementary Fig. 2 Workflow for identification of the *ROS1*-fused gliomas.

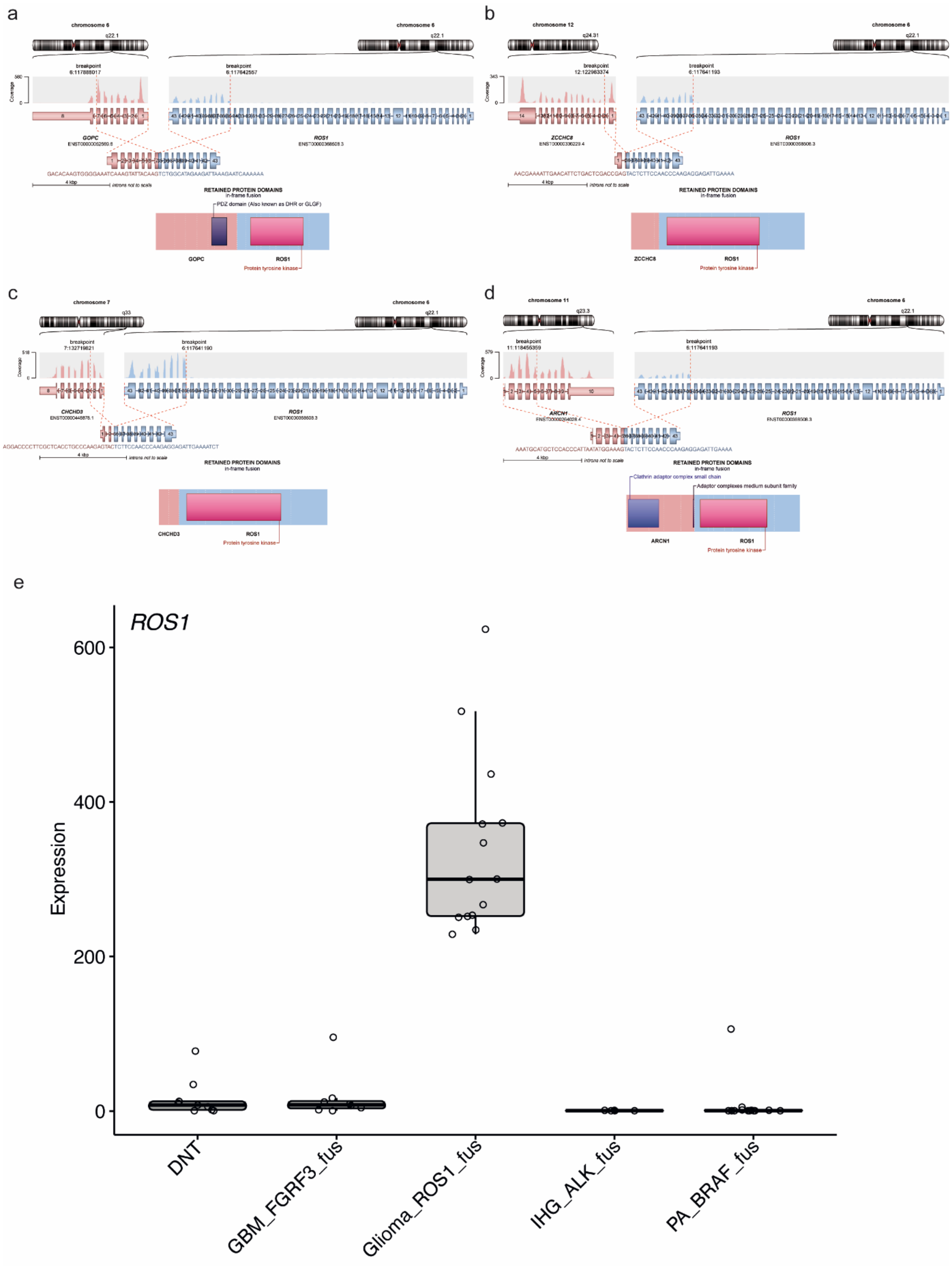

Supplementary Fig. 3 Schematic illustration of the different *ROS1*-fusions detected in the glioma series - *GOPC:ROS1*- (a), *ZCCHC8:ROS1*- (b), *CHCHD3:ROS1*- (c) and *ARCN1:ROS1*-fusion (d). Differential gene expression analysis between *ROS1*-fused cases (Glioma_ROS1_fus; n = 14) and a reference cohort of different low- and high-grade glial/glioneuronal tumors: dysembryoplastic neuroepithelial tumor (DNT; n = 9), glioblastoma IDH-wildtype *FGFR3:TACC3*-fused (GBM_FGFR3_fus; n = 10), infantile hemispheric glioma *ALK*-fused (IHG_ALK_fus; n = 5) and pilocytic astrocytoma *KIAA1549:BRAF*-fused (PA_BRAF_fus; n = 14). *ROS1* is more highly expressed in Glioma_ROS1_fus cases when compared with representative glial/glioneuronal tumors (e).
